# Estimation of Macronutrient Content in Kindergartens Meals: Food Composition Tables or Chemical Analysis?

**Published:** 2014-09-27

**Authors:** Lazarevic Konstansa, Stojanovic Dusica, Bogdanovic Dragan

**Affiliations:** 1 State University of Novi Pazar, Serbia; 2 Public Health Institute Nis; 3 School of Medicine, University of Nis, Serbia

**Keywords:** Macronutrient, Preschool Children, Diet, Kindergarten

## Abstract

***Objective:*** The nutrition of children in kindergartens has a great significance for proper growth and development of children. In order to save time and money, the control of macronutrients content is performed by calculations using food composition tables instead of performing a chemical analysis.

***Methods:*** We examined the macronutrients content of 240 whole day meals using food composition tables and performed chemical analysis of meals to determine adequacy and validity of food composition tables in calculation of macronutrient contents in kindergarten meals.

***Findings:*** We established no correlation (*P*>0.05) between the value of proteins, fats and carbohydrates. Significant difference was established between the average content of proteins (t=2.57; *P*<0.05), and carbohydrates (t=3.20; *P*<0.01), but not with the content of fats in the meals (t=1.26; *P*>0.05) (food composition tables vs chemical analysis).

***Conclusion:*** Until we establish new food composition tables, chemical analysis remains the only valid method for assessment of macronutrients content and energy value of a meal in kindergarten.

## Introduction

Proper nutrition during the period of childhood is the foundation of good health in the adulthood and necessary for proper growth and development of children^[^^1^^]^.

 As a consequence of the modern style of life, characterized by long working day of parents, most of children in developing countries spend 8-12 hours during the day in kindergartens^[^^2^^,^^3^^]^. For this reason the kindergarten diet has an important role in the daily intake of energy and nutrients. *Protein*s from diet are important for growth and development during childhood, and dietary *requirements (g/kg of body weight)* are significantly increased in this period. Dietary fat and carbohydrates are energy sources, but dietary fats are also source of fat soluble vitamins and some fatty acids important for children^[^^4^^]^. 

 There are different methods for estimation of the composition of food: data from printed food composition tables, electronic databases, food industry data and data from scientific literature^[^^5^^]^. In order to save time and money, chemical analysis of meal (often cooked or prepared food) is increasingly rare and calculations by using food composition tables are applied. In few of the studies, nutrient content in kindergarten meals was calculated by food composition tables - studies from Brazil^[^^6^^]^, China^[^^7^^]^, Finland^[^^8^^]^ or by chemical analysis in Croatia^[^^9^^]^ or by both methods as in Poland^[^^10^^]^ and in our study.

 The purpose of this article is to compare results of protein, carbohydrate and fat content in daily kindergartens meals obtained from food composition table and by chemical analysis, and to suggest how to ensure the correct calculation of macronutrients contents in kindergarten meals.

## Subjects and Methods

There are three meals in Niš kindergarten (breakfast, lunch and snack), planned by a nutritionist, a physician and a nurse.

*Use of food composition tables* *.* It was necessary to calculate the amount of consumed food, and then multiply the food intake by the mean nutrient content obtained from the food composition database. Daily amount of food used for preparation of 240 meals is calculated based on the warehouse supply list. Nutrient contents of meals were calculated by Serbian food composition tables^[^^11^^]^.

***Chemical analysis: ***After the collection, the samples of meals in duplicate were transported to the laboratory at 4°C within one hour. They were analyzed for moisture, protein, fat and ash^[^^12^^]^ in the laboratory of the Institute for Public Health, Niš (Serbia). 

 Descriptive statistics (mean, standard deviation) of macronutrient contents (g) was calculated using Microsoft Excel software. Student's t-test and linear correlation was applied to compare the values between two methods.

## Findings

Results for contents of macronutrients in kindergartens meals were similar for fats, but not proteins and carbohydrates when calculated by chemical analysis and food composition tables (Table 1). Significant correlation between proteins, fats and carbohydrates intake obtained from warehouse supply lists and food composition tables were not observed (Table 2).

## Discussion

Data on macronutrients content of meals in the collective nutrition, obtained from food composition tables are mostly informative. 

 Depending on the type of food, nutrient and table or software selected for comparison, there were significant statistical differences between results of laboratory analyses and results calculated though tables and software data^[^^13^^-^^15^^]^. There are several explanations for these results. 

 Firstly, there were differences in macronutrient estimations depending upon the choice of the food composition tables. Some food composition tables are not adequately documented (small number of analyses, inappropriate choice of analysis). Results from 18 laboratories in Europe showed statistically different values for macronutrients in the well-homogenized samples of foods^[^^16^^]^. Using food composition databases from different countries may increase errors in calculation of macronutrient intake, and there is tendency to standardize food composition databases all over the world^[^^17^^]^.

 Secondly, there is a variation in food composition depending on environmental, genetic and processing influences such as feed, soil, climate, genetic resources (varieties/cultivars, breeds), storage conditions, processing, fortification and market share^[^^18^^]^.

**Table 1:** Mean macronutrient content in analyzed samples of meals by chemical analysis and food composition tables

**Macronutrient**	**Food composition tables**	**Chemical analysis**	**t-test**	***P. *** **value**
	**Mean (SD) (g)**	**Mean (SD) (g)**		
**Protein **	40.80 (4.35)	35.0 (6.47)	2.57	0.02
**Fats **	38.18 (6.32)	34.99 (6.07)	1.26	0.2
**Carbohydrates**	151.80 (13.45)	133.93 (13.96)	3.20	0.004

SD: Standard Deviation

**Table 2:** Correlations between macronutrient content in analyzed samples of meals by chemical analysis and food composition tables

**Parameter**	**Coefficient of correlation (r)**	***P. *** **value**
**Protein**	-0.171	0.6
**Fat**	0.290	0.4
**Carbohydrate**	0.010	0.97

 Thirdly, the majority of nutrient databases provide information mainly on the composition of raw foods. Cooking methods, times, temperatures and food preparation habits vary widely by countries, and that can influence the macronutrient content^[^^19^^]^. 

 Fourthly, we don’t have data about food plate waste in kindergartens.

 Contrary to the results of previous studies, Bedogni et al concluded that food composition tables may be used to assess energy, carbohydrate, lipid and protein intake in the military settings^[^^20^^]^. Many food composition tables, including tables used in our study did not consider yield and retention factors. Camilli et al. found that when yield factor and retention factors were applied, the results of the computed nutrients according to food composition tables showed a satisfactory degree for protein and lipid contents^[^^21^^]^.

## Conclusion

It is evident that food composition tables must include the nutrient composition of cooked and prepared foods, as well as yield factor and retention factor. 

 Taking into account the time that preschool children spend in kindergartens and the importance of proper nutrition for child development in that life period, the chemical analysis of foods (dishes) must be applied for situations such as preschool institutional nutrition.
